# Influence of BMI and smoking on IUI outcome with partner and donor sperm

**Published:** 2017-06

**Authors:** S Huyghe, A Verest, A Thijssen, W Ombelet

**Affiliations:** Ziekenhuis Oost Limburg, Genk Institute for Fertility Technology, ZOL Hospitals, Schiepse Bos 6, 3600 Genk, Belgium.; Faculty of Medicine and Life Sciences, Hasselt University, Martelarenlaan 42, 3500 Hasselt, Belgium.

**Keywords:** Intrauterine insemination, smoking, BMI, prognostic factors, life style

## Abstract

There is limited literature on the influence of smoking and BMI on success rates after intrauterine insemination (IUI). As a result of a prospective cohort study we could investigate data from 1401 IUI cycles with partner semen and 1264 IUI cycles with donor semen, primary outcome being clinical pregnancy rate (CPR). Univariate statistical analysis showed significant influence of female BMI on clinical pregnancy in the partner insemination group (CPR of 6,5%, 8%, 16,3% and 9,4% for a female BMI < 20, 20-24.9, 25-29.9 and 3 30, p=0.032), while in the donor group this in uence was not signi cant (CPR respectively 11.1% (BMI< 20), 18.5% (20-24.9), 18.0% (25-29.9) and 14.7% for BMI 3 30). Multivariate analysis through generalized estimating equations (GEE) could not confirm this significant influence of female BMI on fecundity in the partner semen group. For smoking, univariate statistical analysis revealed male smoking to be a negative influence for the clinical pregnancy rate in the partner insemination group (10.9% CPR in couples with male non-smokers versus 5.9% with male partners smoking 1-14 cig/day, p=0.017). After multivariate GEE analysis this result remained significant (p< 0,01). In the donor semen group female non-smoking or smoking less than 15 cigarettes a day turned out to be significantly associated with a higher CPR compared to women smoking more than 15 cigarettes daily (16.8% and 24.5% versus 5.6%, p=0,01). These results were also significant after multivariate GEE analysis (p= 0,047 and p= 0,02).

## Introduction

Compared to IVF (in vitro fertilization), IUI is still a technique with a rather low success rate. The report of the Belgian Register for Assisted Procreation (BELRAP, 2016) revealed a 9.6% CPR per IUI cycle after partner insemination and 14.3% after donor insemination in the year 2014.

Lifestyle factors can influence success rates after assisted reproduction (Homan et al., 2007). In this study we investigated smoking and BMI in a prospective cohort of patients receiving IUI with donor or partner sperm.

## Smoking

The prevalence of smoking amongst women and men in their reproductive years remains high, despite the well-known negative effects on general health. 20% of all Belgians smoked in 2015, of which 17% were daily smokers with an average of 16 cigarettes a day (Stichting tegen kanker, 2015). There is a gap between male smokers (24%) and female smokers (16%), though this gap has been narrowing over the years. 21% of Belgian citizens in their reproductive age (18-44 years) are smokers. In 2015 WHO reports on a European scale a frame of 29% smokers and worldwide 21%, with a bigger gap between male and female (WHO tobacco report, 2015). Different mechanisms of the effect of male and female smoking on fertility have been reported before (Harlev et al., 2015). Therefore we may expect different results on outcome after IVF/ICSI compared to IUI.

### Smoking and male infertility

Harlev et al. (2015) published a review examining the actual effect of smoking on male fertility. Results are often opposing and confusing as there are different outcome parameters used (semen parameters, spermatozoa function, histologic alterations,...), different mechanisms of effect and difficult adjustment for confounders as socio-economic status, alcohol use, etc. 33 studies were reviewed in which the effect of smoking on sperm parameters was investigated, some reported an adverse effect, others showed no significant effect, finally resulting in a lack of conclusive evidence. Two conclusions can be drawn though: firstly there is a more significant association found between smoking and impaired sperm quality amongst the general population compared to the infertile population, secondly there seems to be a dose- dependent relationship with the amount of cigarettes (Harlev et al., 2015).

The different mechanisms of effects on male infertility are not yet fully understood (Harlev et al., 2015). Besides the direct toxic effect on sperm parameters due to oxidative stress (OS), functional impairment in the presence of normal sperm parameters has been described, there is extra OS due to presence of leucocytes in semen of smokers and there is an indirect effect on fertility due to erectile dysfunction, a higher prevalence of varicoeles and altered vesicular and prostatic parameters amongst smokers (Harlev et al., 2015). Other causes include genetic, epigenetic and molecular changes of DNA sperm due to smoking (Harlev et al., 2015).

### Smoking and female infertility

Augood et al. (1998) reported on the association between smoking and the risk of infertility in women including the size of this effect. Twelve observational studies are included in this meta-analysis, the overall value of the Odds Ratio (OR) for risk of infertility in women smokers versus non- smokers is 1,60 with a 95% confidence interval (CI) of 1,34-1,91. To describe a causative relationship between smoking and female subfertility a biological plausibility would be helpful. Women who smoke seem to have an earlier menopause (MacMahon et al., 1982; Baron et al., 1990) and smoking could directly affect tubal or cervical function (Phipps et al., 1987). By calculating a population-attributable risk Augood et al. (1998) concluded that 13% of all infertility cases are probably due to smoking.

### Smoking and outcome of ART

Several meta-analyses and structured reviews have investigated the effect of smoking in patients undergoing IVF-treatment (Klonoff-Cohen et al., 2001; Soares et al., 2008; Waylen et al., 2009). They all described that, compared to non-smokers, smokers required a higher mean gonadotropin doses for ovarian stimulation, had lower peak E2 levels, fewer oocytes retrieved, lower fertilization and implantation rates and lower pregnancy and live- birth rates. Moreover smokers required nearly twice the number of IVF-cycles to conceive.

Concerning the effect of smoking on the outcome of IUI only a very limited number of studies have been published. Farhi and Orvieto (2009) could not demonstrate a difference in clinical pregnancy rate between smokers and non-smokers undergoing IUI. If ovarian stimulation is given, the smokers did require significantly more gonadotropin ampoules in order to achieve a comparable pregnancy rate (Farhi and Orvieto, 2009). In another analysis of the AMIGOS trial using multivariate logistic regression as the statistical tool and after adjustment for covariates, smoking was not significantly associated with pregnancy outcomes after IUI (Hansen et al., 2016). This finding was confirmed in a retrospective analysis performed by Aydin et al. (2013).

## BMI

Obesity is becoming a global pandemic. It has started to replace the more traditional public health concerns, including undernutrition and infectious diseases, as one of the most significant contributors to ill health. According to the WHO’s recommendations, normal adult BMI is between 20-25 kg/m2, an individual with a BMI of 25-29.9 kg/m2 is considered to be overweight, BMI ≥ 30 kg/ m2 is considered to be obese, and BMI ≥ 40 kg/m2 is considered to be severely obese (WHO obesity report, 2016).

The WHO published global estimates in 2014: 39% of adults aged 18 years and over were overweight, 40% of the women and 38% of the male population. Obesity was seen in 13% of world ´s adult population (15% female and 11% male). Between 1980 and 2014 the worldwide prevalence of obesity had doubled.

### Female BMI and fertility

Obesity is known to cause many disorders of female fecundity. Overweight women have a higher incidence of menstrual dysfunction and anovulation. More hormonal changes have been reported such as altered pulsatile GnRH secretion, reduced sex hormone binding globulin, increased ovarian and adrenal androgen, increased LH and increased insulin resistance (Wang et al., 2004).

The value of obesity as a predictor of infertility treatment outcome is controversial. Some investigators have reported a negative effect of a high BMI on pregnancy rates in patients undergoing ART or after controlled ovulation induction (Wang et al., 2004; Aydin et al., 2013; Yavuz et al., 2013). Others have reported no difference in outcomes when comparing obese women with normal-weight women (Dodson et al., 2006; Souter et al., 2011; Isa et al., 2014). One author has even reported an increase in success in obese women compared with normal-weight women (Wang et al., 2004). It has to be said that these studies have been performed in patients undergoing IVF or ICSI.

Some authors have reported on IUI but these results are very conflicting. A significant increase of fecundity from underweight women to obesity has been reported (Wang et al., 2004). Some authors didn’t find any significant influence of BMI on results of IUI (Dodson et al., 2006; Isa et al., 2014; Souter et al., 2011) Aydin et al. (2013) reported a negative effect of an increasing BMI on results in IUI. These conflicting results in studies may be related to methodological discrepancies in the different studies such as the type of treatment, incompletely characterized or unstratified patient heterogeneity, inconsistent definitions of obesity and disregard for the influence of the obese spouse on pregnancy rates.

## Materials and Methods

### Patients

From 01-07-2011 until 30-09-2015 data from 1401 IUI cycles with partner semen and 1264 heterologous IUI cycles were collected prospectively in a tertiary referral infertility centre. Information on smoking status and BMI amongst others was obtained from a questionnaire filled out during the 20 minutes of bed rest following each insemination.

The partner sperm insemination was done in couples who had been trying to conceive for at least one year. Prior to this IUI treatment the women had been through a standard infertility work-up, including medical history, physical examination, pelvic ultrasound, serum hormone assays between day 2 and 4 of the menstrual cycle, ultrasound monitoring of folliculogenesis, ovulation assessment by mid-luteal phase concentrations of progesterone, and a mid-cycle post-coital test in women with regular cycles. Hysterosalpingography (HSG), hysterosonography and/or laparoscopy was used to assess the uterine cavity and presence of at least one patent tube. In case of a suspected tubal or uterine abnormality, a hysteroscopy and/or laparoscopy were performed. In all men at least one or two sperm examinations including analysis of anti-sperm antibodies (ASA) were performed. All couples were tested for HIV, syphilis, hepatitis B and hepatitis C virus before receiving any treatment according to the Belgian guidelines. Couples suffering from unexplained infertility, mild endometriosis, oligo-/ anovulation and moderate male factor infertility, with at least one patent fallopian tube and an Inseminating motile count (IMC) of >1 million after washing procedure were considered eligible for IUI treatment (Ombelet et al., 1997). When the sperm morphology score was 4% or more, IUI was performed with an IMC of ≥0.3 million (Ombelet et al., 2014).

In the donor insemination group all women were either single or lesbian or hetero couples with an azoospermic partner or a partner with a y-linked chromosome genetic disorder. Those women went through the same infertility work-up as described above.

### IUI procedure

Patients were treated in a natural cycle in case of regular cycles. Ovarian stimulation with CC (clomiphene citrate) or hMG (human menopausal gonadotropin) or recFSH (recombinant follicle stimulating hormone) was used in case of unexplained subfertility or oligo-/anovulation. The stimulation protocol was previously described, as well as the sperm examination and preparation (Thijssen et al., 2017a; 2017b). IUI was performed at 20-36h post-hCG. A fraction of the washed motile spermatozoa was inserted up to the uterine fundus and expelled into the cavity. The women remained supine for 20 minutes after the insemination.

### Parameters studied/statistical analysis

In our analysis we looked specifically into the association between male/female smoking (non- smoking, 1-14 cigarettes a day, ≥15 cigarettes a day) and clinical pregnancy rate (CPR being the detection of a gestational sac and foetal heartbeat using ultrasound at 7-8weeks of gestation) and on the other side into the association between BMI (BMI <20 kg/m2, BMI 20-24.9 kg/m2, BMI 25-29.9 kg/m2 and BMI ≥30 kg/m2) and CPR.

To take into account dependency (possibly different cycles for same patient), not an ordinary logistic regression model, but GEE (Generalized Estimating Equations) analyses were used (Liang and Zeger, 1986). This can be seen as an extension of ordinary logistic regression, where the correlation between observations from the same person are taken into account. Firstly the univariate relationship between smoking or BMI and CPR was studied, afterwards a multivariate analysis was run to exclude confounding factors. Apart from smoking and BMI other registered variables were: female and male age (years), primary/secondary infertility, cycle number, ovarian stimulation method, day 0 estradiol and progesterone levels, human chorionic gonadotropin (hCG)-insemination time interval, easy or difficult insemination with difficult being defined as multiple attempts needed to get into the uterine cavity, occurrence of obvious uterine bleeding during or after insemination, fresh and post-thaw sperm quality parameters (i.e. concentration, motility grade A, motility grade A+B, TMSC (total motile sperm count) and IMC) and sperm washing procedure. P-values were considered significant when < 0,05.

## Results

### IUI with donor semen

We included 402 women who received a total of 1264 IUI cycles with frozen donor semen. The mean age of women was 33.4 years (range 20-46 years). Of those 402 women 89.5% were non-smokers, 7.5% smoked 1-14 cigarettes daily and only 3% more than 15 cigarettes a day.

BMI was spread as follows: BMI<20: 9.3%, BMI 20-24.99: 44.7%, BMI 25-29.99: 25.9%, BMI ≥30: 18.8% of female. Outcome results were not available for 11 cycles (0.9%). We noticed 216 clinical pregnancies (17.2% per cycle), 25 biochemical pregnancies (2.0%), 7 extra-uterine gestations (0.6%) and 25 early miscarriages (2,0%).

### IUI with partner semen

A total of 1401 IUI treatments were performed in 556 subfertile couples. The mean age of women was 32.1 years (range 19-47 years). Of those 556 women 85% were non-smokers, 13% smoked 1-14 cigarettes daily and only 2% more than 15 cigarettes a day. Female BMI was <20 in 18%, 20-24.99 in 52%, 25-29.99 in 19% and ≥30 in 11% of women.

The mean age of men was 34.6 years (range 22-63 years). In this group of men 70% were non-smokers, 19% smoked 1-14 cigarettes daily and 11% more than 15 cigarettes a day. Male BMI was < 20 in 2%, 20-24.99 in 43%, 25-29.99 in 43% and ≥ 30 in 11% of men.

Outcome results were not available for 8 cycles (0.6%). We obtained 131 clinical pregnancies (9.4%), 20 biochemical pregnancies (1.4%), 3 extrauterine gestations (0.2%) and 29 early miscarriages (2.1%).

### Smoking

#### IUI with donor semen and female smoking

**Figure 1:**
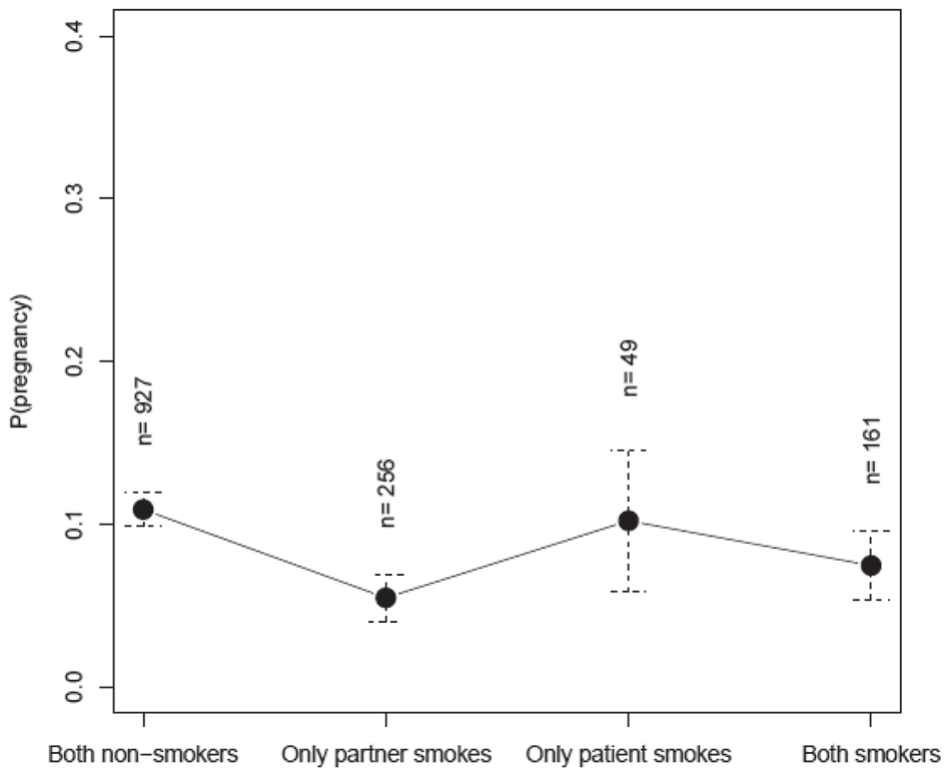
— Probability of pregnancy in function of the couple’s smoking status in couples inseminated with partner’s semen.

Univariate statistical analysis showed that CPR in women smoking 15 cigarettes or more daily was significantly lower compared to women smoking 1-14 cigarettes daily (p=0.01) and there was a nearly significant lower CPR compared with non- smoking women (p=0.05), (Table I, Fig. 1A). After multivariate GEE analysis smoking 15 or more cigarettes daily still decreased CPR significantly compared to women smoking 1-14 cigarettes and to non-smokers (Table II). No significant difference in CPR was observed between women smoking 1-14 cigarettes and non-smokers.

**Table I:** — results from univariate analysis

**Couples with donor semen and female smoking**				
**Smoking (n/day)**	**n (CPR)**	**Total**	**SE**	**P-value**
0 cig	188 (16.8%)	1117	±1.1%	0.088^a^
1-14 cig/day	23 (24.5%)	94	±4.5%	0.052^b^
≥15 cig/day	2 (5.6%)	36	±3.9%	0.014^c^
**Couples with partner semen and smoking of patient vs partner**				
**Smoking patient (n/day)**	**N (CPR)**	**Total**	**SE**	**P-value**
0 cig.	115 (9.7%)	1183	±0.9%	0.417^a^
1-14 cig.	14 (7.9%)	178	±2.0%	0.938^b^
≥15 cig.	3 (9.4%)	32	±5.2%	0.766^c^
**Smoking partner (n/day)**				
0 cig.	106 (10.9%)	976	±1.0%	0,017^a^
1-14 cig.	16 (5.9%)	270	±1.4%	0,126^b^
≥15 cig.	10 (6.8%)	147	±2.1%	0,726^c^

a 0 cig. versus 1-14 cig., b 0 cig. versus ≥15 cig., c 1-14 cig. versus ≥15 cig. n: number of clinical pregnancies; SE: standard error; CPR: clinical pregnancy rate.

**Table II:** — Results from the multivariate GEE analysis- donor semen

**Covariate**	**Parameter estimation (SE)**	**p-value**
Smoking (0 cig.)	1,3461 (0,6777)	**0,047**
Smoking (1-14 cig.)	1,6308 (0,7197)	**0,024**
Smoking (≥15 cig.)	Reference	

Smoking 15 or more cigarettes daily decreased CPR significantly compared to women smoking 1-14 cigarettes and non-smokers when adjusted for confounding factors.

#### IUI with partner semen and female or male smoking

**Figure 3:**
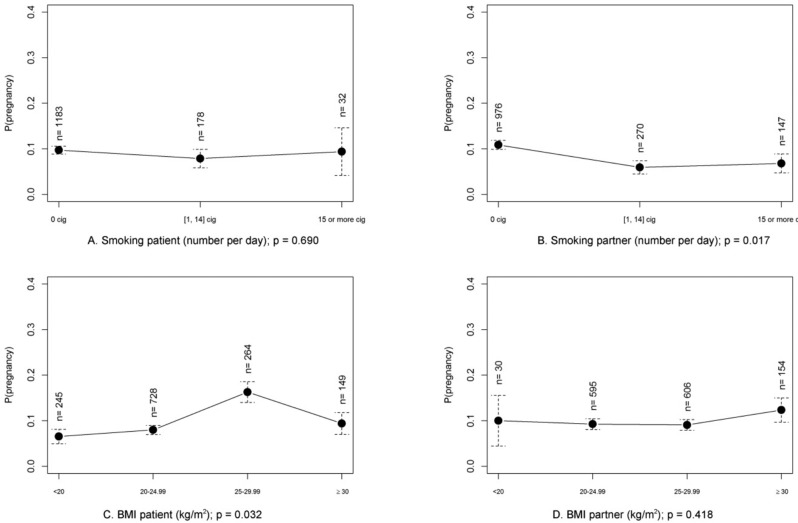
— Probability of pregnancy in function of the couples smoking status (A-B) and BMI (C-D) in couples inseminated with partner’ s semen (results of univariate analysis).

Univariate statistical analysis did not show a difference in CPR with female smoking (p=0.69, figure 3A), but the CPR was significantly affected with the male partner smoking 1-14 cigarettes a day (p=0.01) (Table I, figure 3B), this difference was also significant after multivariate GEE analysis (Table III). However, there was no significant difference in CPR when the partner smoked more than 15 cigarettes a day compared to non-smoking partners. When CPRs were compared in groups with partners smoking 1-14 cigarettes versus partners smoking more than 15 a day, outcome results were not significantly different (p=0.64). Only 32 (2.3%) of women smoked more than 15 cig/day, 147 (10.6%) of men smoked >15 cig/day.

**Table III:** — Results from the multivariate GEE analysis- partner semen

**Covariate**	**Parameter estimation (SE)**	**p-value**
Smoking partner (315 cig.)	0,639 (0,335)	0,089
Smoking partner (1-14 cig.)	0,683 (0,288)	**0,008**
Smoking partner (0 cig.)	reference	

Smoking 1-14 cigarettes daily decreased CPR compared to non-smokers when adjusted for confounding factors.

After multivariate analysis there is also a nearly significant trend of a decreased CPR in groups were both partners smoke versus both partners non- smoking, p=0.052 (Figure 1).

### BMI

#### IUI with donor semen and influence of female BMI

**Figure 2:**
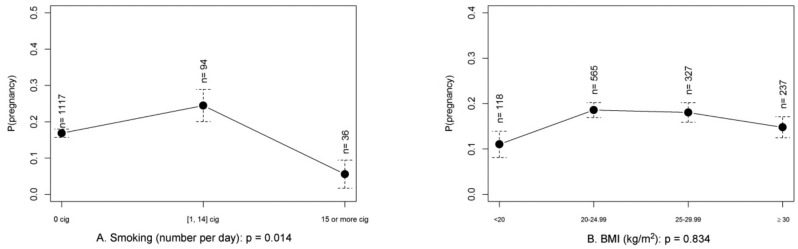
— Probability of pregnancy in function of the patients’ smoking status (a) and BMI (b) in couples inseminated with donor semen (results of univariate analysis).

Univariate analysis of the covariate female BMI in IUI with donor semen showed a non-significant lower CPR when BMI was <20 kg/m2 and ≥30 kg/ m2 (p=0,083) (Table V). When looking into the severe obese (≥35 kg/m2) and morbidly obese (≥40 kg/m2) categories, pregnancy rates were lower, with a CPR of 9.6% (5/52) and 9.1% (1/11), but not significantly different (Fig. 2).

**Table V:** — Univariate analysis

**Couples with donor semen**				
**BMI (kg/m^2^) patient**	**N (CPR)**	**Total**	**SE**	**P-value**
<20	13 (11.1%)	118	±2.9%	0.834
20-24,99	105 (18.5%)	565	±1.6%	
25-29,99	59 (18.0%)	327	±2.1%	
≥30	35 (14.7%)	237	±2.3%	
**Couples with partner semen**				
**BMI patient (kg/m^2^)**	**N (CPR)**	**Total**	**SE**	**P-value**
<20	16 (6.5%)	245	±1.6%	**0,032**
20-24,99	58 (8.0%)	728	±1%	
25-29,99	43 (16.3%)	264	±2.3%	
≥30	14 (9.4%)	149	±2.4%	
**BMI partner (kg/m^2^)**	**N (CPR)**	**Total**	**SE**	**P-value**
<20	3 (10.0%)	30	±5.6%	0,418
20-24,99	55 (9.2%)	595	±1.2%	
25-29,99	55 (9.2%)	606	±1.2%	
≥30	19(12.3%)	154	±2.7%	

BMI: body mass index, n: number of clinical pregnancies, SE: standard error, CPR: clinical pregnancy rate BMI was a continuous variable; therefore p-values represent overall significance levels.

#### IUI with partner semen and influence of BMI

Univariate statistical analysis showed a significantly higher CPR (p=0.032) in patients with a higher BMI up until a BMI of 30 kg/m2 (Fig. 3). However, this result was no longer significant in the multivariate model. Univariate analysis also showed a significant higher female BMI (24.6±4.7) in the group with a positive result for clinical pregnancy. Partner BMI did not significantly influence CPR per cycle (p=0.42) (Table IV).

**Table IV:** — Patient characteristics: Female BMI was significantly higher in the patients with a clinical pregnancy. No significant difference was found with partner BMI.

	Total	Pregnant	Not pregnant	p-value
BMI patient (kg/m^2^)	23,9±4,5 (16,3-43,4)	24,6±4,7 (16,9-42,8)	23,8±4,5 (16,3-43,4)	**0,04**
BMI partner (kg/m2)	25,8±3,5 (16,7-46,1)	26,1±3,3 (19,4-35,4)	25,8±3,5 (16,7-46,1)	0,42

## Discussion

Most observational studies on the influence of lifestyle factors on IUI outcomes are retrospective analyses. This study is interesting and valuable because of the prospective cohort set-up where information about lifestyle was obtained from a questionnaire filled out during mandatory resting time after insemination (Custers et al., 2009).

Another strength is the size of both cohorts. We consider the lack of information on smoking status and BMI of the donors as a limitation.

It is the first study reporting significant associations between male/female smoking and the outcome of IUI. In the few investigations who have addressed lifestyle factors in relation to outcome of IUI, a relationship between smoking and CPR was never described (Aydin et al., 2013; Farhi and Orvieto, 2009; Hansen et al., 2016; Hao Huang et al., 2012). Farhi and Orvieto (2009) only concluded that the smokers did require significantly more gonadotropin ampoules in order to achieve a comparable pregnancy rate.

Looking at male smoking, results presented in this study showed that women with a smoking partner had significantly reduced clinical pregnancy rates. A detrimental effect of smoking on sperm parameters is known, though the complete effect of smoking on fertility remains inconclusive (Harlev et al., 2016).

Female smoking was studied both in the donor and the partner insemination group. In the donor group significantly decreased CPR was observed when women were smoking more than 15 cigarettes a day. This effect was not seen for female smokers in the group of partner semen IUI. This could be due to the small sample size; only 32 cycles were performed in couples where women smoked more than 15 cigarettes a day (2.3%).

The effect of both partners smoking on the outcome of IUI was also studied; results indicating a trend to a decreased CPR compared to the group were both partners are non-smokers. No previous studies were found describing the effect of this cumulative exposure.

Male BMI was not found to be of significant influence in the partner insemination group. For IVF there are multiple studies that showed a negative influence of higher male BMI on sperm morphology, motility and semen volume (Bakos et al., 2011; Håkonsen et al., 2011; Jensen et al., 2004; Keltz et al., 2010;). Female BMI was studied both in the donor and the partner insemination group. In the donor group female BMI showed a tendency towards a lower CPR in the extreme low BMI (<20 kg/m2) and the high BMI (≥30 kg/m2). Female BMI showed to be of significant influence in the univariate statistical analysis in the partner insemination group, since CPRs increased significantly with increasing BMI up until a BMI of 30 kg/m2. However, this result was no longer significant in the multivariate model. These results are largely in accordance with the results described by Wang et al. (2004), who also demonstrated a significant increase in fecundity from underweight to obese women. However, other studies reported no significant differences in pregnancy rates amongst different BMI groups (Dodson et al., 2006; Souter et al., 2011; Isa et al., 2014) or demonstrated a negative effect of high BMI on pregnancy rates (Aydin et al., 2013). When ovarian stimulation is adjusted to overcome the weight effect, it has been reported that CPRs after IUI in obese women are comparable to women with a normal BMI (Souter et al., 2011).

On the other hand, even though the results on BMI are not significant, overweight women should be advised to loose weight because of the obstetrical and foetal problems associated with a high pre-pregnancy BMI. Obstetric complications include hypertensive disorders, gestational diabetes mellitus (GDM), caesarean section (CS) and higher postpartum weight retention (PPWR) (Heslehurst et al., 2008; Poobalan et al., 2009; Nelson et al., 2010). Foetal and perinatal risks include miscarriage, neural-tube defects, heart defects, macrosomia and stillbirth (Callaway et al., 2006; Sarwer et al., 2006).

## Conclusion

Our data indicate a significant association between smoking, male as well as female IUI outcome, more specifically the association with male partner and both partners smoking in the insemination group with partner sperm and the effect of female smoking on the outcome after donor insemination. Therefore a smoking cessation or at least reduction should be advised to all couples undergoing IUI treatment.

For BMI and outcomes of IUI no significant association was found in the multivariate analysis, nevertheless weight loss should be advised to obese women before starting an IUI program to prevent the obstetrical and foetal problems associated with a high pre-pregnancy BMI.
